# Supplementary material for: Comparative analysis of viruses in four bee species collected from agricultural, urban, and natural landscapes
Source: PLoS One. 2020 Jun 12;15(6):e0234431. doi: 10.1371/journal.pone.0234431 (PMC7292363; doi:10.1371/journal.pone.0234431)
Supplement: S1 Fig — Primer pairs species for DWV and IAPV were used separately to amplify RT-PCR products of 194 and 586 bp, respectively. Negative (H2O) and positive controls were included in each run of the RT-PCR. (DOCX) [file pone.0234431.s001.docx]

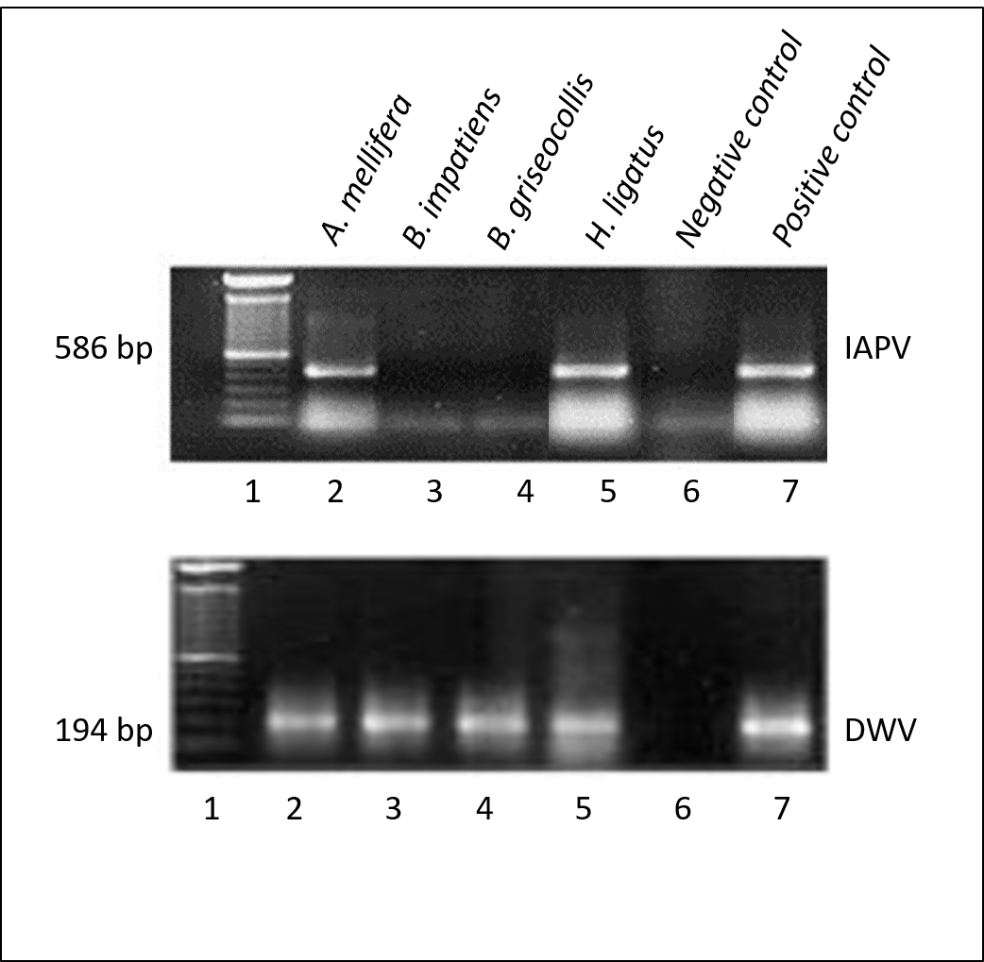


**S1 Fig.** **Detection of DWV and IAPV viruses on *Apis mellifera, Bombus impatiens, B. griseocollis, Halictus ligatus* sampled from urban site in early –mid summer in 2018.** Primers for DWV and IAPV were used separately to amplify RT-PCR products of 194 and 586 bp, respectively. Negative (H2O) and positive controls were included in each run of the RT-PCR.
