# Supplementary material for: Comparative analysis of viruses in four bee species collected from agricultural, urban, and natural landscapes
Source: PLoS One. 2020 Jun 12;15(6):e0234431. doi: 10.1371/journal.pone.0234431 (PMC7292363; doi:10.1371/journal.pone.0234431)
Supplement: S2 Fig — Virus prevalence, or the number of positive detections of BQCV, DWV, IAPV, and or SBV, in pollen loads collected from the legs of foraging honey bees or from in-hive pollen stores collected in early and late summer (2017–2018). (DOCX) [file pone.0234431.s002.docx]

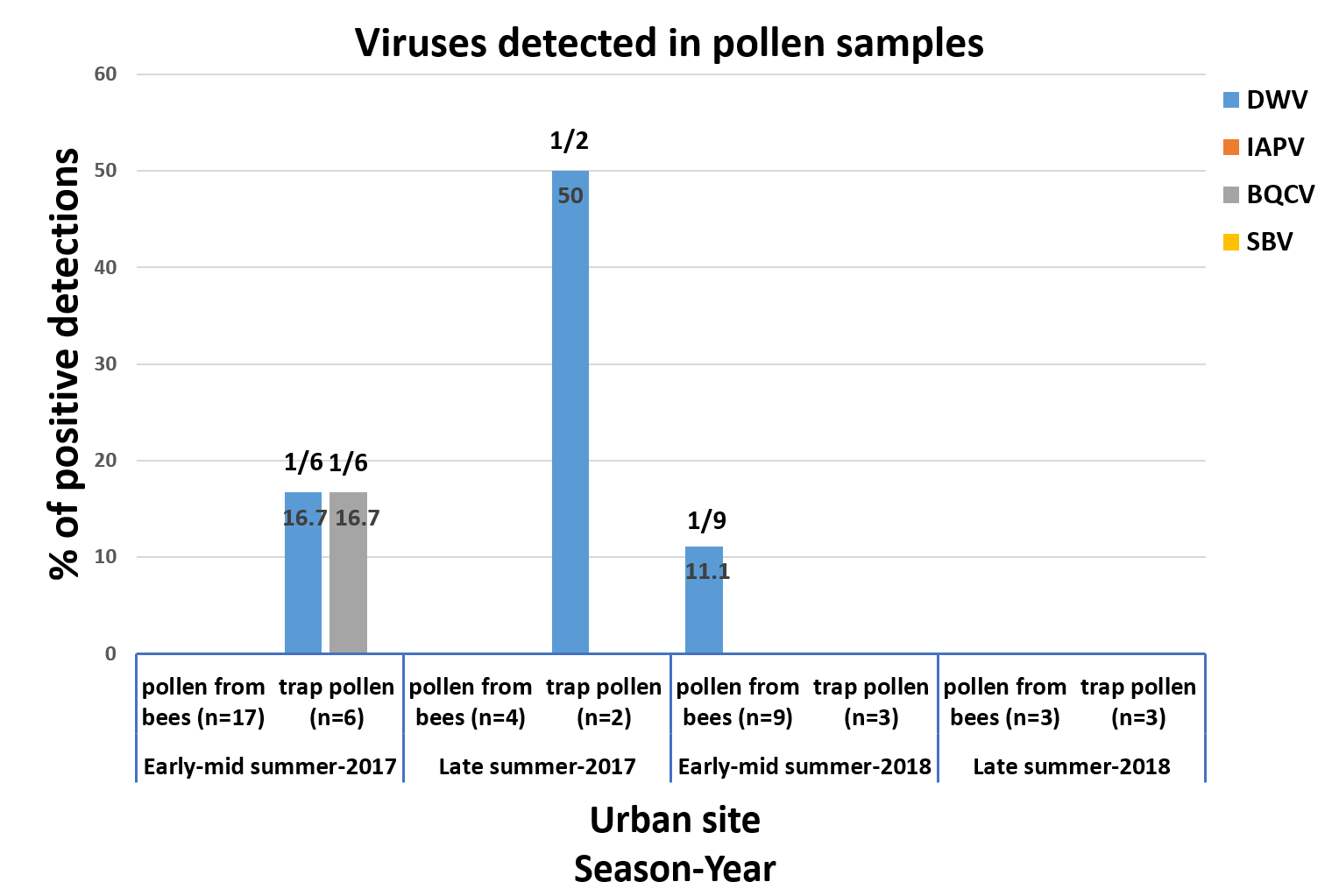


**S2 Fig Viruses detected in pollen samples.** Viral prevalence, or the number of positive detections of BQCV, DWV, IAPV, and or SBV, in pollen loads collected from the legs of foraging honey bees or from in-hive pollen stores collected in early and late summer (2017–2018).
