# Supplementary material for: Comparative analysis of viruses in four bee species collected from agricultural, urban, and natural landscapes
Source: PLoS One. 2020 Jun 12;15(6):e0234431. doi: 10.1371/journal.pone.0234431 (PMC7292363; doi:10.1371/journal.pone.0234431)
Supplement: S1 Table — Sites with “*” denotes larger locations that had multiple distinct collection areas; however, sampling effort was standardized across landscape types and across season. (DOCX) [file pone.0234431.s003.docx]

| **Landscape** | **GPS Coordinates** | **Site name** | **Description** | **Size (ha)** |
| --- | --- | --- | --- | --- |
| Agricultural | 41.16573243, -96.45532608 | UNL Eastern Nebraska Research and Extension Center* | Newly-established (< 2 yrs) pollinator habitat established in field margins of research corn and soybean fields; pollinator-friendly cover crops with legumes. | ⁓3,912 |
|  | 40.73819, -96.75049 | Goat Farm | Organic specialty crops (vegatables and fruit) | ≤1,5 |
|  | 40.94456, -96.54873 | Dixi Farm | Alfalfa hay fields | ≤1,5 |
| Urban | 40.82965755, -96.6549468 | UNL Pollinator Garden and Outdoor Classroom | Diverse pollinator-friendly plantings of both native and cultivated species | ≤1,5 |
|  | 40.80127079,- 96.68185472 | Hamann Rose Garden |  | ≤1,5 |
|  | 40.80216415, -96.68299198 | Sunken Garden |  | ≤1,5 |
|  | 41.23333232, -95.91697454 | Lauritzen Garden* |  | Sites ranged (0.40 - 40.5) |
| Natural or Open space | 40.704873, -96815858 | Lincoln Prairie Corridor* | Tallgrass and shortgrass prairie ecosystem (remenant & restored) | ⁓2,994 |
|  | 40.7312, -95.9268 | Union City Roadsides* | Newly seeded (<2 yrs) with diverse pollinator mixes | ⁓11,007 |

**S1 Table.** Description of the three landscape types (Agricultural, Urban, and Natural/Open) and location information for collection sites from 2017 and 2018. Sites with “*” denotes larger locations that had multiple distinct collection areas; however, sampling effort was standardized across landscape types and across season.
